# Supplementary material for: Complexome profiling of the Chlamydomonas psb28 mutant reveals TEF5 as an early PSII assembly factor
Source: Plant Cell. 2025 Mar 17;37(6):koaf055. doi: 10.1093/plcell/koaf055 (PMC12164590; doi:10.1093/plcell/koaf055)
Supplement: koaf055_Supplementary_Data [file koaf055_supplementary_data.zip › psb28_interactive.zip!/plots/intensity/qualityPlots/peptides/CAM6_Cre02.g108900.t1.2.html]

### Cre02.g108900.t1.2

Trivial name: CAM6  
SeqCoverage [median]: 23.30  
Mass: 19.69 kDa  
  
 

| Sequence | MissedCleavage | Mass [Da] | wtIntensity | muIntensity | Score |
| --- | --- | --- | --- | --- | --- |
| EVYIGLLK | 0 | 933.55 | 276.43 | 0.00 | 64.80 |
| FGLNVTNSK | 0 | 978.51 | 2165.72 | 1258.20 | 140.14 |
| IGVAVLMK | 0 | 829.51 | 524.39 | 0.00 | 77.75 |
| IPSIDDVNK | 0 | 999.52 | 541.24 | 0.00 | 72.48 |
| LYDLINR | 0 | 905.50 | 396.55 | 0.00 | 73.04 |
|  |  |  | 3904.3 | 1258.2 |  |
